# Characterization of Prion Disease Associated with a Two-Octapeptide Repeat Insertion

**DOI:** 10.3390/v13091794

**Published:** 2021-09-08

**Authors:** Nicholas Brennecke, Ignazio Cali, Tze How Mok, Helen Speedy, Genomics England Research Consortium, Laszlo L. P. Hosszu, Christiane Stehmann, Laura Cracco, Gianfranco Puoti, Thomas W. Prior, Mark L. Cohen, Steven J. Collins, Simon Mead, Brian S. Appleby

**Affiliations:** 1 Department of Neurology, Case Western Reserve University & University Hospitals Cleveland Medical, Cleveland, OH 44106, USA; Nicholas.Brennecke@uhhospitals.org (N.B.); mlc11@case.edu (M.L.C.); 2 Department of Pathology, School of Medicine, Case Western Reserve University, Cleveland, OH 44106, USA; ixc20@case.edu; 3 National Prion Disease Pathology Surveillance Center (NPDPSC), Cleveland, OH 44106, USA; 4 MRC Prion Unit at University College London, Institute of Prion Diseases, London W1W 7FF, UK; tze.mok@ucl.ac.uk (T.H.M.); h.speedy@ucl.ac.uk (H.S.); l.hosszu@prion.ucl.ac.uk (L.L.P.H.); s.mead@prion.ucl.ac.uk (S.M.); 5 Genomics England Limited of Dawson Hall, Charterhouse Square, London EC1M 6BQ, UK; 6 Australian National Creutzfeldt-Jakob Disease Registry, The Florey Institute, The University of Melbourne, Melbourne, VIC 3010, Australia; christiane.stehmann@florey.edu.au (C.S.); s.collins@unimelb.edu.au (S.J.C.); 7 Department of Pathology and Laboratory Medicine, School of Medicine, Indiana University, Indianapolis, IN 46202, USA; lcracco@iu.edu; 8 Department of Advanced Medical and Surgical Sciences, University of Campania “Luigi Vanvitelli”, 81100 Caserta, Italy; Gianfranco.PUOTI@unicampania.it; 9 Prion Disease Diagnosis and Surveillance Center (PDDSC), University of Campania “Luigi Vanvitelli”, 81100 Caserta, Italy; 10 Center for Human Genetics Laboratory, Case Western Reserve University & University Hospitals Cleveland Medical Center, Cleveland, OH 44106, USA; thomas.prior@uhhospitals.org; 11 Department of Psychiatry, Case Western Reserve University & University Hospitals, Cleveland, OH 44106, USA

**Keywords:** prion disease, Creutzfeldt-Jakob disease, genetic Creutzfeldt-Jakob disease, genetics, octapeptide repeat insertion

## Abstract

Genetic prion disease accounts for 10–15% of prion disease. While insertion of four or more octapeptide repeats are clearly pathogenic, smaller repeat insertions have an unclear pathogenicity. The goal of this case series was to provide an insight into the characteristics of the 2-octapeptide repeat genetic variant and to provide insight into the risk for Creutzfeldt–Jakob disease in asymptomatic carriers. 2-octapeptide repeat insertion prion disease cases were collected from the National Prion Disease Pathology Surveillance Center (US), the National Prion Clinic (UK), and the National Creutzfeldt–Jakob Disease Registry (Australia). Three largescale population genetic databases were queried for the 2-octapeptide repeat insertion allele. Eight cases of 2-octapeptide repeat insertion were identified. The cases were indistinguishable from the sporadic Creutzfeldt–Jakob cases of the same molecular subtype. Western blot characterization of the prion protein in the absence of enzymatic digestion with proteinase K revealed that 2-octapeptide repeat insertion and sporadic Creutzfeldt–Jakob disease have distinct prion protein profiles. Interrogation of large-scale population datasets suggested the variant is of very low penetrance. The 2-octapeptide repeat insertion is at most a low-risk genetic variant. Predictive genetic testing for asymptomatic blood relatives is not likely to be justified given the low risk.

## 1. Introduction

Prion disease (PrD) can be sporadic, genetic, or acquired. Genetic prion disease (gPrD) accounts for 10–15% of prion diseases and is caused by pathogenic sequence variations in the prion protein gene (*PRNP*) [1]. Most *PRNP* mutations are point mutations but deletions and insertions in the octapeptide repeat region of *PRNP* are reported, referred to respectively as octapeptide repeat deletions (OPRDs) and octapeptide repeat insertions (OPRIs) [2]. The combination and number of repeats in OPRI mutations vary and are thought to contribute to phenotypic variation [1,3,4]. In this study, we sought to characterize the clinical features, histopathology, and molecular typing of the prion protein (PrP) associated with 2-OPRI patients and to determine the risk for Creutzfeldt–Jakob disease (CJD) in carriers of the 2-OPRI variant.

The first case of the 2-OPRI in the setting of CJD was reported in 1993 [5]. The proband died of neuropathologically verified CJD 3 months after presenting with speech and language difficulties. Multiple first-degree family members were carriers of the 2-OPRI variant, including a family member with a long-standing history of dementia.

A second case of 2-OPRI in the setting of dementia was reported in 2000 [6]. The patient presented after years of progressive memory impairment. Genetic studies revealed a 2-OPRI variant with valine homozygosity at codon 129 of *PRNP*. No autopsy was performed. The patient did not have a family history suggestive of PrD.

A third case of 2-OPRI in the setting of dementia was reported in 2004 [4]. The patient developed dementia in their sixth decade of life and then declined over years to a mute state. Autopsy results were not reported. Genetic studies demonstrated heterozygosity at codon 129 of *PRNP*. 

These reports have not provided a conclusion as to the significance of the 2-OPRI genetic variant. While OPRI mutations comprised of four or more repeats are clearly pathogenic in that the majority of mutation carriers develop prion disease in their lifetimes, the pathogenicity of smaller repeats has thus far remained unclear [2]. This paper presents a new collection of 2-OPRI cases in subjects with autopsy-confirmed prion disease and (or) clinical features that satisfy criteria for the classification of probable CJD. The hypothesis at the outset was that 2-OPRI was not a high penetrance mutation but rather a genetic variant that may modify the risk or phenotype of sCJD.

## 2. Materials and Methods

### 2.1. Ethics Approval, Data Collection

Multiple prion disease surveillance centers were queried as to whether or not they had subjects with 2-OPRI variants. Three national prion disease surveillance centers replied that they had subjects with the 2-OPRI variant in their database and agreed to partake in this study. Eight cases were identified from the USA (*n* = 5), the UK (*n* = 2), and Australia (*n* = 1). After an Institutional review board (IRB) review from University Hospitals Cleveland Medical Center (STUDY20201625), the Scotland A Research Ethics Committee (REC code 05/MRE00/63), and the University of Melbourne Human Research Ethics Committee (ethics approval number 1341074), records were reviewed and data were collected on *PRNP* codon 129 polymorphism, family history of PrD, and other neurodegenerative diseases, gender, ethnicity, age at symptom onset, symptom progression, disease duration, brain magnetic resonance imaging (MRI) findings, electroencephalogram (EEG) findings, cerebrospinal fluid (CSF) test results, neuropathologic findings, and prion protein chemistry results (Western blot). CSF testing was conducted as per each country’s protocol [7,8,9,10]. Brain MRIs were evaluated using criteria from Zerr and colleagues [11]. Each case was deemed to represent either definite or probable CJD, depending on the presence or absence of autopsy, respectively. Data from all cases were abstracted using the same instrument. 

### 2.2. Genetic Testing

*PRNP* genetic testing was performed within each surveillance center as previously described [12,13]. *PRNP* was examined for sequence variations and the repeat sequence in the octapeptide region of the gene were noted, if available, as previously described by Goldfarb and colleagues [14]. Codon 129 polymorphism was also described for each case. 

### 2.3. Reagents and Antibodies

For the USA, Proteinase K (PK), phenylmethanesulfonyl fluoride (PMSF), 10× Dulbecco’s Phosphate Buffered Saline (DPBS) were from Sigma–Aldrich (St. Louis, MO, USA). Laemmli Sample Buffer, Tween 20, and 15% Criterion™ Tris-HCl protein gels were from Bio-Rad Laboratories (Hercules, CA, USA). Blocking buffer and Infrared Dye 800CW goat anti-mouse IgG (1 mg/mL) were from LI-COR Biosciences (Lincoln, NE, USA). Polyvinylidene fluoride (PVDF) membrane (Immobilon-FL) was from EMD Millipore (Billerica, MA, USA). The monoclonal 3F4 antibody (Ab) to PrP, epitope 106–110, was used [15,16]. The UK followed previously published protocols [12].

### 2.4. Brain Tissue

Brain autopsy of USA patients with 2-OPRI (*N* = 5), controls sCJDMM1 (*N* = 2), and sCJDMM2 (*N* = 1) was performed at the NPDPSC in Cleveland, Ohio. The autopsy of the 7-OPRI case was carried out at the Prion Disease Diagnosis and Surveillance Center (PDDSC) in the Department of Advanced Medical and Surgical Sciences, University of Campania “Luigi Vanvitelli” (Caserta, Italy) [17,18]. The molecular (Cali I.) and histopathological (Cali I. and Cohen M.L.) study of US cases were done at the NPDPSC. Brain regions examined by Western blotting included the frontal cortex (2-OPRI cases 1–5 of Table 1, 7-OPRI, sCJD controls), occipital cortex (2-OPRI cases 1 and 3), and cerebellum (2-OPRI cases 1–5). Brain autopsy and histopathological examination of the UK patient were performed at the National Hospital for Neurology and Neurosurgery on Queen Square, London. The molecular strain typing and Western Blot were performed on frozen frontal cortex at the MRC Prion Unit at UCL [19].

### 2.5. Histology and PrP Immunohistochemistry

For the USA cases, the formalin-fixed brain was treated as previously described [18]. Tissue was deparaffinized, rehydrated, and immersed in Tween 20-Tris buffered saline. The endogenous peroxidase was blocked by the Envision Flex Peroxidase Blocking Reagent (Dako North America Inc., Carpinteria, CA, USA) for 10 min, which was followed by several washes. Sections were immersed in hydrochloric acid (1.5 mmol/L), microwaved for 15 min, and incubated with purified 3F4 Ab (1:1000) for 1 hour. Sections were washed and incubated with Envision Flex/HRP polymer for 30 min (Dako). The Envision Flex DAB (Dako) was used to show the immunostaining. Brain tissue blocks from the only UK-autopsied case were from 1985 and not suitable for modern immunocytochemistry.

### 2.6. Preparation of Brain Homogenates and Detergent-Insoluble Fractions

For the USA cases, frozen brain material was homogenized in 1× Lysis buffer (LB100) (100 mM NaCl, 0.5% Nonidet P-40, 0.5% sodium deoxycholate, 10 mM EDTA, 100 mM Tris-HCl, pH 8.0) [20]. The resulting 10% BH was subjected to low speed centrifugation (1000× *g*) for 5 min at 4 °C and the supernatant (S1) was collected. Aliquots of S1 from the five USA cases were further subjected to high speed centrifugation (100,000× *g*) for 1 h at 4 °C to generate the detergent-insoluble disease-associated PrP (PrP^D^), which was resuspended in 1× LB100 pH 8.0. Detergent insoluble PrP^D^ samples were obtained from the frontal cortex of (i) 2-OPRI with methionine (M) homozygosity at codon 129 (129MM) and PK-resistant PrP^D^ (resPrP^D^) type 1 (2-OPRI-MM1; *n* = 3), (ii) 7-OPRI with 129MM genotype and resPrP^D^ type 1 (7-OPRI-MM1; *n* = 1), and (iii) sCJDMM1 (*n* = 2). 

### 2.7. Proteinase K Digestion and Western Blot (WB) Analysis

For the USA cases, S1 were treated with 10 units/mL (U/mL) PK (48 U/mg specific activity, 1 U/mL equal to 20.8 µg/mL PK) at 37 °C for 1 h. The reaction was stopped by addition of PMSF (3 mM). Each sample was mixed with an equal volume of 2× Laemmli buffer and boiled at 100 °C for 10 min. Proteins were loaded onto a 15% Tris-HCl Criterion™, 8.7 cm-long gels. Western blot analysis was performed as previously described [21,22]. The Ab 3F4 was used at 1:10,000 dilution. Membranes were developed by the Odyssey near-infrared imaging system (LI-COR Biosciences) and densitometric analysis was performed with the Odyssey software V3.0 (LI-COR Biosciences). Most of the WB were performed in triplicate. Typing of resPrP^D^ of cases obtained at the NPDPSC was assessed using the “Parchi et al.” molecular classification [23,24]. For clarity, in this manuscript, the UK resPrP^D^ was converted to the same typing classification, according to “Parchi et al.”.

### 2.8. Penetrance Estimation

Three largescale population genetic databases were queried for the 2-OPRI allele: gnomAD v2.1.1 (USA), gnomAD v3 (USA), and 100,000 Genomes Project (UK). The total allele counts used for penetrance calculations from the gnomAD v2.1.1 and v3 datasets were 279,320 (range 247,484–279,320) and 142,740 (range 141,382–142,740), respectively, while the total allele count for the 100,000 Genomes Project was 66,670. The upper ends of the total allele counts from gnomAD v2.1.1 and v3 were used in the calculations. Within these 3 datasets, assertion of unaffected status is secure in the 100,000 Genomes Project, as both 2-OPRI alleles originate from individuals within non-neurological cohorts. However, this is not the case for the gnomAD datasets as variant–phenotype data are not routinely available and confirmed to not be shareable following official enquiries. The list of contributing cohorts to the gnomAD datasets include Alzheimer’s disease, migraine, and psychiatry cohorts, which cannot be deemed strictly non-neurological.

The central estimate of disease penetrance is equal to the proportion of individuals with the disease who have the genotype (no. of 2-OPRI alleles found in CJD cases ÷ all *PRNP*-sequenced CJD cases) multiplied by the prevalence of the disease (lifetime risk of CJD (0.02%)), and divided by the frequency of the genotype in the general population (no. of 2-OPRI alleles found in a specific dataset ÷ number of *PRNP* alleles sequenced in that particular dataset) [25,26]. The Wilson Interval is best used to calculate the 95% confidence interval here as the central estimate is close to zero.

$$Penetrance=\frac{lifetime\;CJD\;risk\;x\;frequency\;of\;2OPRI\;allele\;in\;CJD\;cases}{frequency\;2OPRI\;allele\;in\;normal\;population}$$

$$Penetrance\;=\frac{lifetime\;CJD\;risk\;x\;(2OPRI\;alleles\;in\;CJD\;cases\;÷\;no.of\;sequence\;PRNP\;CJD\;alleles)}{\left(2OPRI\;alleles\;in\;genetic\;dataset\;÷\;no.of\;sequenced\;PRNP\;alleles\;in\;genetic\;dataset\right)}$$

## 3. Results

### 3.1. Clinical Features and Genetics

Eight cases were compiled, and clinical data is shown in Table 1. All cases were of white race. The median age at onset was 75 years (range 58–84 years, mean 71 years ± 8.1). The age at disease onset was known for six cases and mean age at onset differed significantly between methionine homozygotes and heterozygotes at codon 129 (64.3 ± 5.5 and 78.0 ± 5.3, respectively, *t*-test, *p* = 0.036). The median disease duration was 7 months (range 2–21 months). All cases with known dates of onset had an illness duration of 7 months or less, with the exception of one case of probable CJD that had an illness duration of 21 months. Family history is relevant because highly penetrant mutations typically show an autosomal dominant inheritance pattern in pedigrees, whereas low penetrance variants might only rarely cause CJD in relatives. Family history of neurodegenerative disease was noted in three cases. No cases had a family history of prion disease. The clinical phenotype of the 2-OPRI cases was heterogenous but marked by typical CJD features, including dementia, ataxia, pyramidal signs, myoclonus, and visual symptoms. One case demonstrated periodic sharp wave complexes on an electroencephalogram. Brain MRIs were obtained in all but one case and were consistent with PrD: all MRIs demonstrated cortical hyperintensity on diffusion weighted imaging (DWI), while three of seven cases demonstrated hyperintensity in the basal ganglia. All but one case demonstrated a positive 14-3-3 in the CSF. The mean CSF tau value was 12,402 pg/mL (high tau levels, e.g., >1500 pg/mL, are suggestive of prion disease) [8]. All cases tested by real time quaking-induced conversion (RT-QuIC) were positive. All cases demonstrated two repeats in the octapeptide region of *PRNP*. Repeat sequences were available in seven cases: Most cases demonstrated repeats in R2-R2 (Table 2). Five cases were homozygous for methionine at codon 129. Three cases were methionine-valine heterozygous.

**Table 1:** Clinical presentation of 2-OPRI cases.

Case	Origin	Diagnosis	Codon 129 Genotype	resPrP^D^ Type ^a^	Age at Onset (Years)	Disease Duration (Months)	Gender	14-3-3 Protein	Tau (pg/mL) ^b^	RT-QuIC	PSWC on EEG	MRI c/w CJD	Family History	Clinical Phenotype
1	US	Definite	MM	1	67	2	Male	Pos.	17,727	NA	NA	Yes	None	Slurred speech, then a fulminant course including cognitive and cerebellar symptoms and myoclonus
2	US	Probable	MM	1	NA	NA	Male	Pos.	8848	NA	NA	Yes	None	NA
3	US	Definite	MM	1	68	7	Female	Pos.	7990	NA	Yes	Yes	Mother with several year slowly progressive dementia in her 60s, thought to be AD	Absence-like episodes, followed 5 months later by cognitive symptoms, personality change, and myoclonus
4	US	Probable	MV	1–2	84	3	Female	Unk.	Unk.	NA	NA	Yes	None	Myoclonus, unilateral weakness/spasticity, late cognitive symptoms
5	US	Definite	MM	1–2	NA	NA	Female	Pos.	15,046	Pos.	NA	Yes	None	NA
6	UK	Probable	MV	NA	76	10	Male	Neg.	Unk.	Pos.	No	Yes	Sister with 2-year history of Alzheimer’s dementia starting at age 89; sister with mild dementia in her 80s.	Gait ataxia, followed by cognitive symptoms, visual hallucinations, and myoclonus
7	UK	Definite	MM	1	58	7	Male	Unk.	Unk.	NA	No	NA	Unknown	Right hand paresthesia followed by unilateral weakness/spasticity
8	AU	Probable	MV	NA	74	21	Female	Pos.	Unk.	Pos.	No	Yes	Brother with 5-year history of dementia in his 60s.	Visual disturbances, followed by gait disturbance, Parkinsonian features, apraxia, and visuospatial difficulties

^a^ Parchi et al. classification [24]; ^b^ high tau levels, e.g., >1150 pg/mL, are suggestive of prion disease [8]; resPrP^D^: PK-resistant disease-associated PrP; Codon 129 polymorphism in cis with the 2-OPRI mutation is underlined if known. US: United States; UK: United Kingdom; AU: Australia; RT-QuIC: real-time quaking-induced conversion; Pos.: positive; Neg.: negative; Unk.: unknown; PSWC: periodic sharp wave complexes; NA: not available; AD: Alzheimer’s disease.

### 3.2. Histopathology and Immunohistochemistry

**Figure 1:**
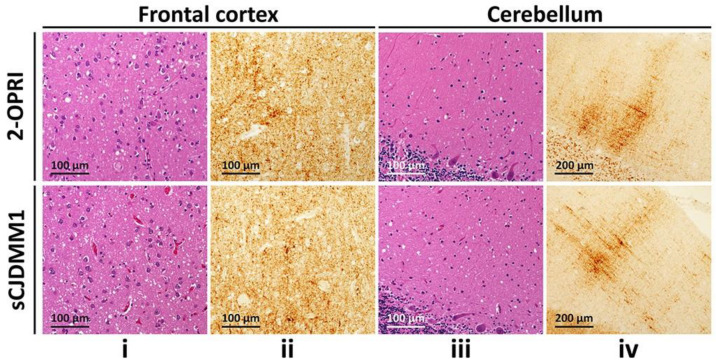
Histological determination of 2-OPRI and sCJDMM1: (**i**,**iii**): Hematoxylin-eosin staining. (**ii**,**iv**): PrP immunohistochemistry. (**i**,**iii**): Fine spongiform degeneration. (**ii**,**iv**): Diffuse PrP immunostaining (**ii**,**iv**) with the typical “brush stroke-like” deposits in the cerebellar molecular layer (**iv**); antibody: 3F4.

Neuropathology was available in six out of eight cases. Four out of five cases demonstrated classic spongiform degeneration (SD) and reactive astrocytosis on hematoxylin and eosin staining with a synaptic pattern of PrP deposition on immunohistochemical examination (cases 1–3 and 7, Table 2) (Figure 1). These histopathological features matched those associated with sCJDMM(MV)1. case 5 demonstrated the sCJDMM1-2 histotype with a minor type 2 component, characterized by large vacuoles and perivacuolar PrP deposition in the temporal cortex. case 4, carrying the 129MV genotype and resPrP^D^ type 1–2, showed mild SD with small and large vacuoles, and diffuse and coarse PrP deposits within the neocortex. No features suggestive of gPrD, such as filamentous PrP deposits in the subcortical white matter or “striped” cerebellar PrP staining characteristic of some OPRIs, were observed in the autopsied brains [27,28].

### 3.3. Characterization of Detergent-Insoluble and PK-Resistant PrP^D^ (resPrP^D^)

**Figure 2:**
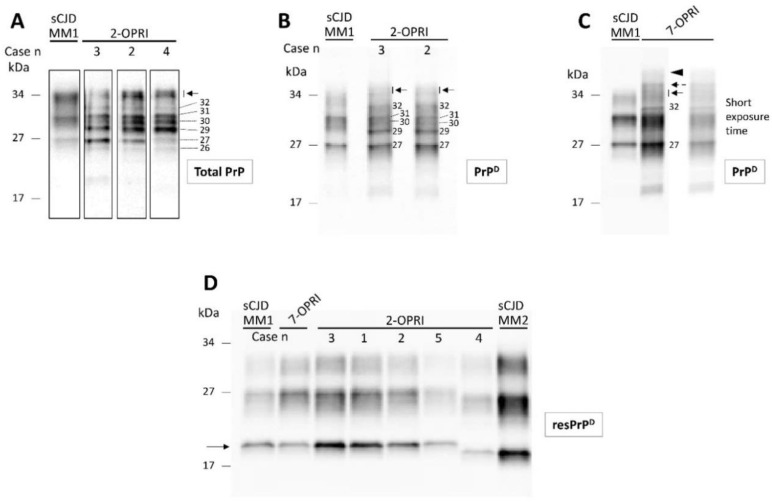
Western blot profiles of total PrP, detergent-insoluble PrP^D^ and resPrP^D^: Samples harvested from the cerebral cortex were probed with the anti-PrP antibody 3F4. (**A**): Total PrP showing a complex PrP profile in 2-OPRI cases but not in sCJDMM1. The approximate molecular size of each band is indicated by the numbers on the right; arrow: PrP doublet of ~34–35 kDa. (**B**): Western blot (WB) profile of detergent-insoluble PrP^D^ harvested from 2-OPRI resembles that of total PrP except for the absence and weak presence of the ~26 and ~30 kDa fragments, respectively. (**C**): Insoluble PrP^D^ WB profile of a 7-OPRI features a sharp band of ~32 kDa, a PrP^D^ triplet in the ~34–36 kDa region, and a PrP^D^ smear of ~42–44 kDa, whereas the PrP^D^ region of ~31 to 27 kDa resembles that of sCJDMM1. (**D**): The un-glycosylated isoform of resPrP^D^ (arrow) in 2-OPRI-MM1 (cases 1–3), 2-OPRI-MM1-2 (case 5), and 7-OPRI-MM1 migrates to ~20 kDa, matching the gel mobility of resPrP^D^ type 1 (sCJDMM1). The un-glycosylated resPrP^D^ of 2-OPRI-MV1-2 (case 4) migrates to ~19 kDa, thus matching the gel mobility of sCJDMM2 resPrP^D^ type 2.

Total PrP of the 2-OPRI (cases 2–4) showed several fragments spanning from ~26 to ~35 kDa. Thus, the PrP profile of 2-OPRI differed markedly from that of sCJD, which consisted of three major bands corresponding to di- (~33 kDa), mono- (~31 kDa), and un-glycosylated (~27 kDa) PrP isoforms (Figure 2A). Following high-speed centrifugation, we generated detergent-insoluble PrP^D^. Western blot analysis demonstrated that most of the PrP fragments of the 2-OPRI cases are detergent-insoluble and that most of them (e.g., 29, 30, 32 kDa, and a doublet of ~34–35 kDa) are not detectable in sCJD. (Figure 2B). Furthermore, the 2-OPRI PrP^D^ profile was different from that observed in a 7-OPRI case. Notably, the ~34–35 kDa doublet appeared as a triplet in the 7-OPRI case, while a band with the molecular weight of ~42–44 kDa was not detected in 2-OPRI (Figure 2C) [18]. Following digestion with PK, the Western blot profile of resPrP^D^ from the OPRI and sCJD cases became virtually indistinguishable [18,29,30].

### 3.4. Estimation of 2-OPRI Penetrance

Interrogation of a number of available large-scale population genetic datasets identified a total of sixteen 2-OPRI alleles in presumably unaffected individuals. Within these 16 alleles, 6 (5 exomic, 1 genomic) were from gnomAD v2.1.1 (USA), 8 from gnomAD v3 (USA), and 2 from the 100,000 Genomes Project (UK) [31]. Of the 14 alleles from the gnomAD datasets, 7 were non-Finnish Europeans, 4 were Finnish Europeans, 1 was South Asian, and 1 was African and 1 Latino/admixed American; 8 were male and 6 were female. Age range data was available for 5 of 6 gnomAD v2.1.1 cases only, with 2 cases in the 55–60 years range, 2 cases in the 60–65 year range, 1 case in the 65–70 years age range, and unknown for 1; age range data for gnomAD v3.1.1 is available for 2 of 8 cases, with 1 case in the 65-70 years age range and 1 case in the 70-75 years age range. Estimation of 2-OPRI penetrance and 95% CI using a Bayesian approach and Wilson interval, respectively, revealed an extremely low penetrance of 0.3% and below, and upper bounds of 95% CI below 2% [25]. Specifically, the estimated penetrance by leveraging the gnomAD v2.1.1 was 0.34% (95% CI 0.08, 1.46), gnomAD v3 was 0.13% (95%CI 0.03, 0.51), and, for the 100,000 Genomes Project, it was 0.24% (95%CI 0.03, 1.75).

## 4. Discussion

The landscape of gPrD genetic variants and clinicopathological phenotypes is heterogeneous. Penetrance varies between *PRNP* mutations and some genetic variants affect disease risk and phenotypes but are not truly pathogenic (e.g., codon 129 polymorphism). As genetically-based treatment trials are on the horizon, estimating the penetrance of *PRNP* mutations is increasingly important [32]. In cases of *PRNP* OPRI variants, those with four or less extra repeats tend to resemble a CJD phenotype, while those with larger repeat insertions often suffer for longer durations and (or) a Gerstmann–Sträussler–Scheinker syndrome phenotype [1]. Little is known about prion disease associated with the lowest number of repeats in the octapeptide region.

In this study, we present clinicopathological and genetic data on eight 2-OPRI cases from three different countries. A variety of typical features were reported in cases with clinical data, and the median illness duration was 7 months, all of which were consistent with a classic CJD phenotype. Case 8 was an outlier with regard to protracted disease duration, passing away 21 months after symptom onset. This case was not autopsy confirmed, but met the criteria for probable CJD. Cases 6 and 8, which were both heterozygous at PrP codon 129, had the longest disease duration. Although data on PrP^D^ type was not available for these two cases, the long disease duration suggests the presence of type 2 or mixed types 1 and 2 [33]. Age at disease onset was typical for sCJD. The length of OPRI in combination with *PRNP* codon 129 genotype has been used to explain variation in age at onset, as observed in a previously published OPRI case series [4]. We found a similar association in our 2-OPRI series, with codon 129 methionine homozygotes having a younger mean age at onset compared to heterozygotes. Only three cases had a family history of neurodegenerative disease, with all three being non-suggestive of a prion disease phenotype, and no cases had a family history of prion disease.

Neuropathological findings were consistent with what is observed in sCJD. Cases 1–3, 5, and 7—all methionine homozygous at PrP codon 129—showed the sCJDMM(MV)1 histotype. A minor sCJDMM2 component was observed in the temporal lobe of case 5 [22]. In case 4, neuropathological changes were characterized by diffuse and coarse PrP deposition and resembled the sCJDMV2C + 1 histotype [33]. Although the wet tissue of the cerebellum was not available, Western blot analysis of the same brain region detected resPrP^D^ type 1, which reinforces the diagnosis of MV2C + 1.

The PK-untreated PrP Western blot profile from the five U.S. 2-OPRI cases significantly differed from that of sCJDMM1 and other sCJD subtypes (Figure 2). Furthermore, the total PrP and detergent-insoluble PrP^D^ of the 2-OPRI cases were similar except for variations in the prevalence of PrP fragments. Thus, although 2-OPRI cases and sCJD cases share histopathological features, they show distinct PrP profiles only in absence of proteolytic digestion.

Estimations of 2-OPRI penetrance by leveraging large-scale population genetic datasets in the USA and UK uniformly resulted in very low lifetime risks of between 0.13 and 0.24%, with upper bounds of 95% CI of 1.75%. For comparison, the estimated lifetime risk of developing prion disease in the United States is 1:6239 or 0.016%, suggesting an ~8–15-fold increase in risk above background [34]. The relative abundance of 2-OPRI alleles in these datasets produced narrow 95% CIs, thus avoiding the potential pitfall of underestimating the 95% CI upper bound that afflicts novel sequence variants of a very low frequency such as singletons [25,26]. Of note, since large-scale datasets such as gnomAD and the 100,000 Genomes Project comprise data produced using short-read sequencing technologies, with read lengths of up to 150 bp, it is likely that 2-OPRI allele frequency in these datasets, and as such in the general population, is underestimated. While the normal octapeptide repeat region is 123 base pairs long, 2-OPRI alleles are 171 base pairs long, meaning that short sequencing reads are unable to fully resolve the spectrum of OPRI alleles present. 2-OPRI is different from highly penetrant mutations of *PRNP*, which make CJD inevitable or highly likely in a lifetime; rather, the 2-OPRI mutation increases the risk of CJD, but only to the extent that the lifetime risk for carriers is approximately 1 in 500.

Most of the pathogenic mutations seen in inherited prion disease occur within residues 89–231 of PrP [35,36], which form the relatively protease-resistant core of the disease-associated form of PrP^D^, and are sufficient to support prion replication and the development of pathology [37]. The octapeptide repeat insertions or deletions are the exception [35,36]. The mechanism by which the octapeptide repeat region influences the formation of PrP^D^ is unclear, especially since this section of the protein is unfolded and highly mobile in the normal cellular form of PrP (PrP^C^) and is rapidly digested by proteinase K in PrP^D^ [38]. PrP is known to bind a range of divalent metal cations, including Cu^2+^ and Zn^2+^, with sub-stoichiometric quantities of Cu^2+^, causing self-association of the prion protein in vitro [39], suggesting that Cu^2+^ may play a role in controlling oligomerisation in vivo. The histidine residues of the octapeptide repeats, together with two histidines at residues 96 and 111, contribute to its Cu^2+^-binding properties. Thus, an expanded or contracted octapeptide region may affect PrP metal co-ordination and folding, or affect a metal-dependent function of PrP^C^, with 4-OPRI or greater being required to significantly affect PrP metal co-ordination and pathology.

This report has several limitations. Clinical data were absent in two cases, limiting conclusions on effect of the *PRNP* codon 129 genotype on variation in age at onset. Neuropathological data were absent in two cases. The conclusions are limited by sample size. Ascertainment bias may arise from the sole focus in obtaining detailed clinical *PRNP* sequencing and autopsied brain material from those presenting to prion disease surveillance centers. The dichotomous phenotypes of CJD and long-duration dementia syndromes are well-described even within the same pedigrees in larger OPRIs, most notably in 4- and 5-OPRI families [40,41]; furthermore, the penetrance in these pedigrees are notoriously incomplete, and ages of onset can be extremely variable. In our 2-OPRI case series, three patients had immediate blood relatives with long-duration neurodegenerative disease presumed to be non-prion disease etiologies such as Alzheimer’s disease, but none of them underwent *PRNP* sequencing, and no autopsied brain material was examined to exclude prion pathology. If PrD neuropathology was identified in these relatives with long-duration neurodegenerative disease syndromes, this line of evidence would have influenced our conclusions.

Despite these limitations, the multiple lines of evidence presented here indicate that 2-OPRI observed in conjunction with CJD demonstrates typical clinicopathological features of sCJD, but a different biochemical profile. The relative abundance of 2-OPRI in multiple large-scale population genetic datasets and a lack of a family history of prion disease in these cases is reassuring. The extremely low penetrance of close to 0%, with upper bounds of 95% CIs of below 2%, suggest that 2-OPRI is, at most, a very low-risk variant. There may also be non-*PRNP* genetic variants that affect penetrance [42]. Predictive clinical genetic testing for asymptomatic blood relatives is not likely to be justified, given the extremely modest increased lifetime risk. However, ongoing research of prion disease patients with 2-OPRI genetic variants and their family members will be helpful for informing and elucidating the pathogenic contribution from this rare genetic variant.

## 5. Conclusions

The 2-OPRI genetic variant does not appear to be a pathogenic mutation but may slightly increase risk of developing sporadic CJD. Cases of prion disease with the 2-OPRI genetic variant resemble sporadic CJD.

## Figures and Tables

**Table 2:** Molecular features and histotype of 2-OPRI cases.

Case Number	Codon 129 Genotype	Repeat Sequence ^a^	resPrP^D^ Type ^b^	sCJD Histotype ^b^
1	MM	R1-R2-R2-**R2-R2**-R3-R4	1	MM(MV)1
2	MM	R1-R2-R2-R3-**R2-R3**-R4	1	MM(MV)1
3	MM	R1-R2-R2-**R2-R2**-R3-R4	1	MM(MV)1
4	MV	R1-R2-R2-**R2-R2**-R3-R4	1-2	MV2C + 1 ^c^
5	MM	R1-R2-R2-R3-**R2a-R2a**-R4	1	MM1-2^c^
6 (UK)	MV	R1-R2-R2-**R2-R2**-R3-R4	NA	NA
7 (UK)	MM	R1-R2-R2-R3-**R2a-R2a**-R4	1	MM(MV)1.
8 (AUS)	MV		NA	NA

^a^ As defined by Goldfarb et al. [14]; ^b^ Parchi et al. classification [24]; c Minor sCJDMM2 component affecting the temporal cortex; Codon 129 polymorphism in cis with the 2-OPRI mutation is underlined if known. resPrP^D^: PK-resistant disease-associated PrP; MM: methionine homozygosity; MV: methionine/valine heterozygosity; NA: not available.
